# Type D Adipsia with Severe Hypernatremia: A Unique Presentation of an Extensive Intracranial Germinoma

**DOI:** 10.31662/jmaj.2022-0194

**Published:** 2023-03-24

**Authors:** Ana Lilia Peralta-Amaro, Mitzi Gabriela Márquez-Vargas, José Carlos González-Molinero, María de Jesús Cruz-Gómez, Juan José Gómez-Piña, Carlos Alberto Hernández-Jiménez, Tania Stefania Cabrera-Castillo, Abihai Lucas-Hernández, Alfonso Carus-Sánchez, Marco Antonio Alvarado-García, Leopoldo Cruz-González

**Affiliations:** 1 Internal Medicine Department, Hospital de Especialidades Centro Médico Nacional “La Raza”, Instituto Mexicano del Seguro Social, Mexico City, Mexico; 2 Division of Postgraduate Studies, Universidad Nacional Autónoma de México, Mexico City, Mexico; 3 Neurology Department, Hospital de Especialidades Centro Médico Nacional “La Raza”, Instituto Mexicano del Seguro Social, Mexico City, Mexico; 4 Radiology Department, Hospital de Especialidades Centro Médico Nacional “La Raza”, Instituto Mexicano del Seguro Social, Mexico City, Mexico

**Keywords:** intracranial germinoma, hypernatremia, adipsia

## Abstract

Intracranial germ cell tumors are uncommon brain tumors; germinoma is the most common tumor in children and young adults, and the most common regions affected are pineal gland and suprasellar region. Germinomas of the suprasellar region are accompanied by endocrine alterations, with adipsia being a rare presentation. Here, we present the case of a patient with an extensive intracranial germinoma whose initial presentation was adipsia, without any other endocrinological alteration, with development of severe hypernatremia and unusual manifestations derived from it, such as deep vein thrombosis, myopathy with rhabdomyolysis, and neurological axonal damage.

## Introduction

Intracranial germ cell tumors (icGs) are rare neoplasms that represent between 0.5% and 2.1% of primary brain tumors in children and adolescents; two-thirds are germinomas, and the rest are nongerminomas. Clinical manifestations of icGs depend on the location and size of the tumor. In addition, icGs are considered highly curable brain tumors ^(1)^.

## Case Report

**Figure 1.**
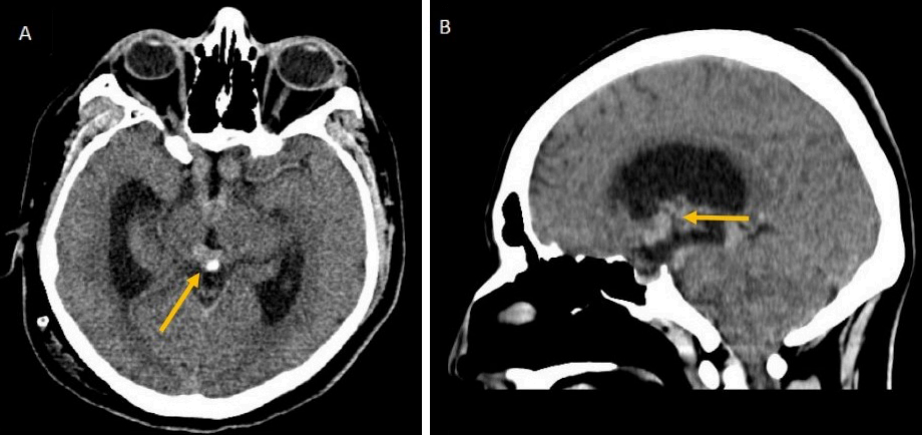
Brain computed tomography findings. A. Axial nonenhanced computed tomography (NECT) scan shows typical findings of pineal germinoma with a well-demarcated slightly hyperdense tumor engulfing the calcified pineal gland. B. Sagittal NECT scan shows a lobulated hyperdense mass with different locations, suprasellar, infundibular, and tumor spread, in the lateral ventricles and third ventricle with hydrocephalus.

A 22-year-old man came to our hospital due to a six-month-long generalized decrease in muscle strength. Regarding important background, nine months ago he presented with decreased visual acuity of the right eye and then hydrocephalus with intracranial hypertension, for which a ventriculoperitoneal bypass valve was placed. Next, brain computed tomography (CT) showed a lobulated hyperdense tumor with different locations: suprasellar, infundibular, and pineal, with hydrocephalus (Figure 1). Three months later, he presented with thrombosis of the iliac vein and left common femoral vein, and he received rivaroxaban 20 mg q24h, without any medical follow-up.

On physical examination, we found a pale patient with dehydrated oral mucosa, and the neurological examination showed the following: visual acuity of the right eye 20/200 and the left 20/100, no papilledema, bitemporal hemianopsia, and Parinaud’s syndrome (downward conjugate gaze in primary position with bilateral limited supraversion, dilated pupils with hyporeflexia, and upgaze convergence nystagmus). Moreover, he presented with decreased muscle tone and strength (4/5 thoracic limbs and 3/5 pelvic limbs), decreased tendon reflexes, and ataxic gait.

Further, his laboratory tests showed anemia, elevated lactate dehydrogenase, hypernatremia, and elevated creatine phosphokinase. During his hospitalization, hypernatremia was confirmed (sodium 170-182 mEq/L) with a serum osmolarity of 353.4 mOsm/kg. Urinary osmolarity and urinary specific gravity were normal (Table 1). Further, in the laboratory tests of nine months ago, the hypernatremia also stood out (sodium 185 mEq/L).

**Table 1.** Laboratory Tests.

	Value	Reference Value
**Blood chemistry**		
Glucose, mg/dL	103.1	70-105
Creatinine, mg/dL	1.26	0.5-0.9
Albumin, mg/dL	4.1	3.4-5
Alanine aminotransferase, UI/L	25.6	13-40
Total Bilirubin, mg/dL	0.42	0.2-1
Lactate dehydrogenase, UI/L	463	180-250
		
**Serum electrolytes**		
Sodium, mEq/L	172	136-145
Potassium, mEq/L	4.3	3.5-5
Creatinine phosphokinase, UI/L	11,236	24-195
		
**Complete Blood Count**		
Hemoglobin, g/dL	10.2	12-18
Hematocrit, %	36.1	37-52
Leukocyte, K/μL	9.5	4.5-10
Neutrophils, K/μL	8.3	3.0-6.9
Lymphocyte, K/μL	0.8	0.6-3.4
Platelets, K/μL	144	150-400
Prothrombin time, s	15	11-15
Partial thromboplastin time, s	33	30-40
		
**General urine test**		
Color	Light yellow	Light
Density	1.010	1.005-1.030
Nitrites	Negative	Negative
Glucose	Negative	Negative
		
**Tumor markers**		
Carcinoembryonic antigen, ng/mL	0.38	0-2.5
Alpha-fetoprotein, ng/mL	2.93	< 40
Carbohydrate antigen 19.9, UI/mL	5.1	< 30
		
**Hormone test**		
Total Triiodothyronine T3, ng/dL	121.17	64-181
Thyroxine T4, ng/dL	0.57	0.53-1.34
Thyroid stimulating hormone, μUI/mL	2.38	0.4-4.94
Prolactin, ng/mL	16.59	2.5-17
Adrenocorticotropic hormone, pg/mL	42.82	0-46
Cortisol, μg/dL	8.24	5-25
Growth hormone, ng/mL	0.12	0.05-1
		
**Osmolality**		
Serum osmolality, mOsm/kg	353.4	280-300
Urine osmolality, mOsm/kg	350	50-1200

The patient did not manifest thirst and did not present with polyuria. In addition, he was treated with hypotonic solutions with maximal sodium decreasing to 160 mEq/L.

**Figure 2.**
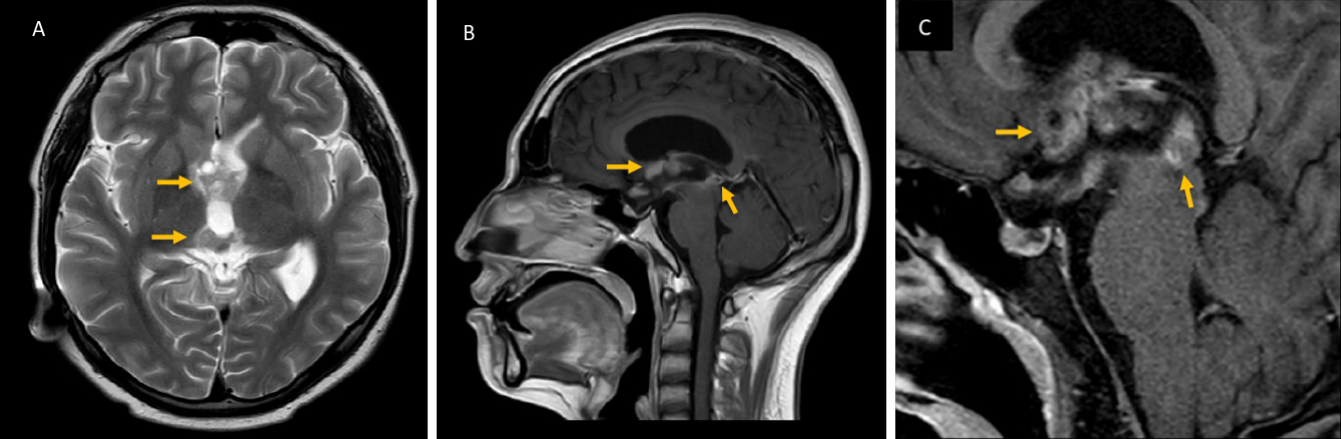
Brain magnetic resonance imaging (MRI) findings. A. T2WI MRI Axial Lesions common isointense and hyperintense in the third ventricle with multiple cysts in the germinoma. B. Sagittal T1WI C+ MRI shows homogeneous suprasellar enhancement of the hypothalamus and chiasmatic region, with extension to the pineal gland through the floor of the third ventricle. C. Sagital T1WI C MR. Large suprasellar lesion with obstructive hydrocephalus with the appearance of an enlarged lateral ventricle and flattening of the fornix.

To assess the current characteristics of the lesion observed in the previous brain CT, a brain magnetic resonance imaging with contrast was performed. It showed a hypothalamic-chiasmatic tumor with extension to the pineal gland through the floor of the third ventricle (FTV) (Figure 2). The biopsy of the tumor reported germinoma. Testicular ultrasound showed no evidence of tumor, and the CT scan ruled out metastases. Hormonal function of anterior pituitary and blood levels of β-human chorionic gonadotropin and alpha-fetoprotein were normal (Table 1). Next, electromyography and conduction velocities showed muscle membrane instability and a sensorimotor polyneuropathy with axonal damage.

The final diagnosis was adipsia with severe hypernatremia secondary to primary extragonadal germinoma of the hypothalamus with extension to the chiasmatic region and pineal gland through the FTV. The patient received radiotherapy and sequential chemotherapy with ifosfamide and cisplatin, with which he presented with a complete response.

## Discussion

Endocrinological alterations can be part of the presentation of germinomas of suprasellar region (SSR) or present as a complication of treatment. The most frequently reported endocrine disorder is diabetes insipidus (DI), which is characterized by polydipsia and polyuria (urinary volume > 300 ml/hr or >4 liters/day, with urinary osmolarity <200 mOsm/kg and urinary density <1.005) ^(1), (2), (3)^.

Adipsia is a rare endocrine disorder which is caused by damage to the circumventricular organs of the anterior hypothalamus where the thirst osmoreceptors are located. Adipsic patients do not perceive osmotic dehydration and therefore do not respond with an adequate increase in fluid intake, leading to the development of hypernatremia. The hypernatremia can be complicated by thrombotic episodes, rhabdomyolysis, and peripheral neurological damage ^(2)^. Next, adipsia can be divided into the following four types: Type A adipsia, also known as essential hypernatremia or partial DI, is characterized in that patients are thirsty and secrete vasopressin, which allows them to be protected from severe hypernatremia. Type B adipsia, is where patients demonstrate a normal osmoregulatory set point, but thirst and vasopressin responses to a hyperosmolar challenge are insufficient to preserve this set point. Type C adipsia, is where a widespread central destruction occurs leading to a complete absence of vasopressin release and thirst response in the presence of an osmotic stimulus, and such a combination is termed adipsic DI (ADI). Currently, three cases of icG with ADI as part of their presentation exist, one of them bifocal ^(3), (4), (5)^. Lastly, Type D adipsia, an extremely rare condition, is where the osmoregulation of vasopressin release is intact, but the thirst response is absent ^(2)^.

Like Alhassan MA, et al. ^(6)^, we considered that our patient demonstrated the integrity of the vasopressin osmoregulation, since he presented with normal urinary osmolarity. In addition, DI was ruled out due to the absence of polyuria, including masked DI, since polyuria did not occur after intravenous hydration and no other signs of hypocortisolism were found. Therefore, the possibility exists that our patient’s adipsia was Type D, although in our setting, performing osmoregulation tests was not possible, as in other reported cases of Type D adipsia ^(7)^.

Finally, no other case was found in the literature in which adipsia was the only endocrine disorder in the initial presentation of an intracranial germinoma.

## Article Information

### Conflicts of Interest

None

### Author Contributions

All authors participated in the acquisition and interpretation of data, as well as in the writing, revision, and final approval of the manuscript. All authors meet the ICMJE authorship criteria.

### Consent for Publication

Consent to publish was obtained from the patient.
